# The Effects of Vibro-Tactile Biofeedback Balance Training on Balance Control and Dizziness in Patients with Persistent Postural-Perceptual Dizziness (PPPD)

**DOI:** 10.3390/brainsci13050782

**Published:** 2023-05-11

**Authors:** Claudia Candreia, Heiko M. Rust, Flurin Honegger, John H. J. Allum

**Affiliations:** 1 Department of ORL, Cantonal Hospital, CH-6016 Luzern, Switzerland; claudia.candreia@luks.ch; 2 Department of Neurology, University of Basel Hospital, CH-4031 Basel, Switzerland; heiko.rust@usb.ch; 3 Department of ORL, University of Basel Hospital, CH-4031 Basel, Switzerland; flurin.honegger@usb.ch

**Keywords:** PPPD, postural control, dizziness, balance deficit, vestibular rehabilitation, vibro-tactile balance training, trunk sway

## Abstract

Background: Patients with persistent postural-perceptual dizziness (PPPD) frequently report having problems with balance control. Artificial systems providing vibro-tactile feedback (VTfb) of trunk sway to the patient could aid recalibration of “falsely” programmed natural sensory signal gains underlying unstable balance control and dizziness. Thus, the question we examine, retrospectively, is whether such artificial systems improve balance control in PPPD patients and simultaneously reduce the effects of dizziness on their living circumstances. Therefore, we assessed in PPPD patients the effects of VTfb of trunk sway on balance control during stance and gait tests, and on their perceived dizziness. Methods: Balance control was assessed in 23 PPPD patients (11 of primary PPPD origin) using peak-to-peak amplitudes of trunk sway measured in the pitch and roll planes with a gyroscope system (SwayStar™) during 14 stance and gait tests. The tests included standing eyes closed on foam, walking tandem steps, and walking over low barriers. The measures of trunk sway were combined into a Balance Control Index (BCI) and used to determine whether the patient had a quantified balance deficit (QBD) or dizziness only (DO). The Dizziness Handicap Inventory (DHI) was used to assess perceived dizziness. The subjects first underwent a standard balance assessment from which the VTfb thresholds in eight directions, separated by 45 deg, were calculated for each assessment test based on the 90% range of the trunk sway angles in the pitch and roll directions for the test. A headband-mounted VTfb system, connected to the SwayStar™, was active in one of the eight directions when the threshold for that direction was exceeded. The subjects trained for 11 of the 14 balance tests with VTfb twice per week for 30 min over a total of 2 consecutive weeks. The BCI and DHI were reassessed each week and the thresholds were reset after the first week of training. Results: On average, the patients showed an improved balance control in the BCI values after 2 weeks of VTfb training (24% *p* = 0.0001). The improvement was greater for the QBD patients than for the DO patients (26 vs. 21%), and greater for the gait tests than the stance tests. After 2 weeks, the mean BCI values of the DO patients, but not the QBD patients, were significantly less (*p* = 0.0008) than the upper 95% limit of normal age-matched reference values. A subjective benefit in balance control was spontaneously reported by 11 patients. Lower (36%), but less significant DHI values were also achieved after VTfb training (*p* = 0.006). The DHI changes were identical for the QBD and DO patients and approximately equal to the minimum clinical important difference. Conclusions: These initial results show, as far as we are aware for the first time, that providing VTfb of trunk sway to PPPD subjects yields a significant improvement in balance control, but a far less significant change in DHI-assessed dizziness. The intervention benefitted the gait trials more than the stance trials and benefited the QBD group of PPPD patients more than the DO group. This study increases our understanding of the pathophysiologic processes underlying PPPD and provides a basis for future interventions.

## 1. Introduction

The term, persistent postural-perceptual dizziness (PPPD), describes a chronic functional vestibular (perceptual) disorder characterised by a sensation of dizziness, non-spinning vertigo and/or unsteadiness which is present for over 3 months on at least 15 days of each month and is aggravated by upright posture [1]. PPPD is used to describe various previous concepts of functional dizziness such as phobic postural vertigo [2], visual vertigo [3], space motion discomfort [4], and chronic subjective dizziness [5] when these are present chronically. The triggering physiological event for PPPD is usually an acute vestibular loss resulting from vestibular neuronitis or benign paroxysmal positional vertigo, although anxiety itself and other traumatic life events can be a trigger [1,6]. Normally, alternative systems of movement control that are not dependent of vestibular information, for example, proprioceptive and visual reflexes, are activated to compensate for the vestibular loss until normal function is restored [6]. In PPPD, it is thought that this normal compensation is replaced by a constant uncompensated state which is typically provoked by upright posture, visual stimuli, and motion [6].

PPPD is a frequent condition observed in tertiary reference centres for dizziness and balance and in general practice [7]. However, because the concept of PPPD was only introduced recently by a committee of experts from the Bárány society [1], few definite statistics on the prevalence of this disorder are available. The prevalence of PPPD that is available from the two largest studies with 21,000 and 9200 patients visiting a tertiary balance clinic with the chief complaint of dizziness and vertigo, indicated for both studies, that 20% of the patients had PPPD, the second-largest diagnosis group [8,9].

Significant insights into the symptoms, physiological mechanisms, and treatment routes for PPPD have been reported over the last decade [10]. Several seminal publications, for example, Dieterich and Staab [11], have described the effects of PPPD on patients with and without sensory deficits which might lead to a deficit in balance control.

The definition of PPPD suggested by Staab et al. [1] clarifies the clinical definition. Nonetheless, the clinical presentation of PPPD patients can be heterogeneous with approximately 50% of patients having an impaired postural control [12], often displaying functional patterns of balance control [13,14] in addition to differences in perceived balance control and body sway [15]. Another common comorbidity in patients with PPPD is vestibular migraine [16]. Given the heterogeneous nature of PPPD, the question arises which treatment procedure is the most effective in reducing dizziness and accompanying deficits in balance control.

One standard psychological procedure for reducing dizziness is to use cognitive behavioural therapy (CBT) to reduce the effect of counteracting personality disorders, such as phobic anxiety, on dizziness. CBT is a psycho-social intervention that is used in psycho-somatic medicine to reduce symptoms of various mental health conditions, primarily depression and anxiety disorders, and is helpful for conditions other than PPPD, e.g., harmful alcohol use; see Tan et al. [17]. For dizziness, CBT is often combined with vestibular physiotherapeutic rehabilitation (VR) to improve balance control. For example, Schmid et al. [18] and Edelman et al. [19] established that CBT improved dizziness and related physical symptoms as well as avoidance behaviours. Furthermore, Holmberg et al. [20,21] showed that the positive effects of CBT for their patients with dizziness, but normal clinical balance control, lasted up to a year. Mahoney et al. [22] also showed that the effect of CBT lasted for at least 6 months. The common CBT approach described above, of psychotherapeutic intervention for PPPD patients with and without balance disorders accompanying dizziness, raises the question whether the presence of a balance disorder affects the treatment outcome. Schmid et al. [12,23] investigated this question in PPPD patients with dizziness only (DO) and those having a quantified balance deficit (QBD), as defined in Section 2 (Methods), in addition to dizziness.

The results of Schmid et al. [12,23] indicated that CBT combined with VR brought significant reductions in the psychologic measures of PPPD patients as measured with the “Brief Symptom Inventory” questionnaire (BSI) introduced by Derogatis and Melisaratos [24]. Specifically, phobic anxiety, obsessive/compulsive, and aggressive/hostile behaviours were improved in the DO patients and highly correlated with reductions in the Dizziness Handicap Inventory (DHI) scores (R = 0.74). However, only phobic anxiety was improved in the QBD patients, leading to a lower correlation with the DHI scores (R = 0.55). Because of the relationship between anxiety and vestibular-based perception of body sway [25,26], as induced by a fear of falling, a decrease in body sway was expected to occur with the reduction in anxiety. However, the results of the Schmid et al. studies [12,23] showed that balance control was not changed for the DO patients and had a modest 8% improvement in the QBD patients [12,23] following CBT with VR. This 8% improvement is in marked contrast to the 20% and larger improvements in balance control obtained by multiple sclerosis (MS) patients after 2 weeks of training with vibro-tactile feedback (VTfb) of trunk sway [27]. VTfb is a technique used to provide the wearer with information on when a trunk sway threshold for a specific sway direction has been exceeded. Vibrators mounted in a headband or belt are then active. For further details, see the methods Section 2.5). The result with the MS patients [27] suggested to us a possibility of improving balance control in PPPD patients with VTfb of trunk sway. Furthermore, we hypothesised that improved balance control in the form of a reduction in trunk sway might also lead to an improved effect of dizziness on the DHI scores.

When working with the MS patients, we relied on the previous work in our lab on the carry-over effect of VTfb after training with VTfb to improve balance control when tested without VTfb [27]. This is different from an on-line effect for which, for example, vestibular loss patients demonstrate changes in muscle activation patterns influencing balance control [28]. The reliance on a carry-over effect for the improvement in balance control has the advantage that the patient does not have to wear the feedback device constantly, and the device does not have to be programmed to recognise that a stance or gait task with different sway amplitudes is being performed by the user. We hypothesised that the VTfb training would help the PPPD patients learn to recalibrate their own falsely perceived sensory signal strengths, thereby improving their balance control, presumably in a similar manner to how the visual feedback was used by San Pedro Murillo et al. [15] to demonstrate a difference between the perceived and actual body sway. Thus, the main questions we aim to address in this report are as follows: Does VTfb training improve the balance control of PPPD patients and, based on our previous work [12,23], is this improvement different for DO and QBD PPPD patients? The second main question we aimed to address was whether the VTfb training reduced the effect of dizziness on daily living as recorded by the DHI scores.

## 2. Methods

### 2.1. Setting

This study was carried out at the Departments of ORL, University Hospital Basel and Cantonal Hospital, Luzern, Switzerland, using the data of patients with persistent dizziness who were treated as described below. For the current retrospective study, patients were divided into 2 groups dependent on whether they had pre-VTfb therapy, pathological balance control or not during stance and gait tests, with respect to the balance of the healthy age-matched controls [29,30]. Patients provided informed consent for the use of their data for scientific purposes. This study (number 2014-026, Amendment 5) was approved by the local ethical committee responsible for the University Hospital Basel and Cantonal Hospital, Luzern: Ethics Committee for North–Central Switzerland (German abbreviation, EKNZ).

This report is based on data collected from 23 patients between October 2020 and November 2022. The sample size of no less than 9 patients per group was based on a power analysis (80%) that revealed that trends of improvement in the balance control of QBD patients, as noted in our previous publication [12], that would reach statistical significance (*p* ≤ 0.05).

### 2.2. Study Groups of Subjects

The 23 subjects included in this study were accepted into our therapy program if they suffered from persistent dizziness for at least 3 months, during at least 15 of 30 consecutive days, and their neurotology and neurology test results indicated that they fulfilled the exclusion criteria described below. As indicated in the table of demographic data (Table 1), patients’ dizziness was either constantly present or episodic. All participants were examined for vestibular and neurological deficits before the VTfb treatment with a battery of video-oculographic oculomotor (optokinetic nystagmus and smooth pursuit eye tracking) and vestibular tests (caloric canal paresis and rotating chair or video head impulse test) which tested for the presence of a central or peripheral vestibular deficit [31]. Vibration sense in the feet was used to test for a lower-leg polyneuropathy among other tests [32]. Patients were excluded if they fulfilled any of the following 7 exclusion criteria: (i) no central vestibular deficit in oculomotor tests, (ii) no physical inability to participate in the physiotherapy exercises, (iii) no visual deficit affecting balance control, (iv) no evidence of a medium to severe form of depression, (v) no indication of a lack of motivation or ability to cooperate with medical personnel (e.g., lack of sufficient German, English, or Italian language skills), (vi) no severe comorbid psychiatric disorders (e.g., schizophrenia), and (vii) no orthopaedic disorders that would have affected balance control. Most patients were transferred to our tertiary hospitals from ear, nose, and throat; neurology; and psychiatric practices as well as general practitioners in Basel or Luzern and the surrounding area of north–west Switzerland.

### 2.3. Dizziness Handicap Inventory

The subjective impression of the impact of dizziness on daily life (a primary outcome of this study) was scored by the Dizziness Handicap Inventory (DHI) questionnaire. The DHI, introduced by Jacobson and Newman [34], assesses the subjective impression of the impact of daily life impairments caused by dizziness. We primarily used a German translation of the DHI, which was validated by Volz-Sidiropoulou et al. [35], and set this impact as minor, medium, or major based on whether the DHI score was above 0, 30, or 60, respectively. Depending on the language abilities of the patient, we also used the original English version [34] or an Italian version of the DHI [36]. The DHI has 25 questions, provides a global score, and captures 3 aspects of impairment, including emotional, functional, and physical. Examples of the questions for these 3 aspects are, respectively, as follows: “Because of your dizziness or unsteadiness problem, do you feel frustrated?”; “Because of your problem, do you have difficulty reading?”; “Do quick movements of your head increase your problem?” Participants could choose to answer in 3 ways as follows: yes (score 4), sometimes (score 2), or no (score 0). Thus, the maximum global score was 100.

### 2.4. Balance Control Index (BCI)

We used the same techniques described previously [29,30] to measure balance control during stance and gait tasks and to establish whether balance control was, as defined by the BCI, within normal limits or not. For this purpose, lower-trunk sway was recorded with a gyroscope system (SwayStar™ Balance Int. Innovations, Iseltwald, Switzerland). The stance and gait tests comprised 14 tasks as follows: (tasks 1 and 2) standing on 2 legs with eyes open and with eyes closed on a normal surface; (tasks 3 and 4) standing on 1 leg with eyes open and with eyes closed on a normal surface; (task 5) walking 8 tandem steps on a normal surface while looking at the feet; (tasks 6 and 7) standing on a foam surface on 2 legs with eyes open and with eyes closed; (task 8) standing on a foam surface on 1 leg with eyes open; (task 9) walking 8 tandem steps on a foam surface while looking at the feet; (tasks 10, 11, and 12) walking 3 m while rotating the head from side-to-side or walking 3 m while flexing and extending the neck, or with eyes closed; (task 13) walking up and down a set of stairs with 2 steps up and 2 steps down; and (task 14) walking over 4 low barriers 24 cm in height and spaced 1 m apart.

For the two-legged stance tasks, participants were asked to stand still on a firm or foam surface in a normal, comfortable standing position, with the lateral borders of the feet hip-width apart, and arms hanging alongside the trunk. The foam surface was 10 cm high, with a 44 cm width and 204 cm long and had a density of 25 kg/m^3^. For eyes-open stance tasks, patients were asked to focus on a black filled circle, 10 cm in diameter, placed at eye height, 5 m away. During one-legged tasks, patients chose their better leg to stand on. Stabilizing their raised leg against the standing leg was not allowed. All walking tasks (with eyes closed, with head rotating or pitching up and down, or walking over 4 low barriers) were measured over 3 m. During all tasks, patients were asked not to talk and a spotter stood next to them to aid them in case of a loss of balance control. For the assessment sequences, each task was performed once and only repeated once if a loss of balance occurred. In training sequences, each task was repeated 4 times. Tasks were stopped if the patient lost balance, for example, needing to take a step during 2-legged stance trials, or if the non-stance foot touched the floor during 1-legged stance trials, or when the task was completed at 20 s for stance trials. Only data prior to any loss of balance was analysed. During the training and the assessment sequences, patients were requested to sway as little as possible. When the patients received VTfb of trunk sway, they were requested to try to avoid activating the vibro-tactile transducers and to move away from the direction indicated by the feedback if the VTfb was activated.

The test results for the assessment sequences were combined to yield a *Balance Control Index* (*BCI*) for every patient. Every BCI was then compared to the age-matched normal reference values [37]. Measures used in the BCI were the peak-to-peak range of angular displacement and velocity in the roll and pitch directions from each assessment trial used by the BCI (see below) as well as trial durations. The measures were combined into a single value, the Balance Control Index or BCI [30], as follows:

(1) $$\mathrm{BCI}=2\;\;*\;\;{s2ecf}_{pv}+{tan8}_{ra}+1.5\;*\;{w3ec}_{pv}+20\;*\;{w3ec}_{dur}+1.5\;*\;{w3hp}_{pv}+12\;*\;{stairs}_{ra}$$

where s2 stands for standing on 2 legs, ec for eyes closed, f for foam, *pv* for peak-to-peak pitch velocity, tan8 for 8 tandem steps, *ra* for peak-to-peak roll angle, w3 for walking 3 m, *dur* for duration, and hp for head pitching. A patient was assumed to have an objectively determined balance deficit if the patient’s BCI was greater than the normal age-matched upper 95% value for the BCI. Based on this BCI criterion, 2 groups of PPPD patients were defined as follows: those with a so-defined balance deficit whom we termed as patients with an objectively quantified balance deficit (QBD), and patients with normal BCI values who were classified as having normal balance control and dizziness only (DO). Normal BCI values and task roll and pitch amplitudes were based on previously recorded data from healthy control subjects (including data from Allum et al. [37] and Hegeman et al. [30]). We used, for this purpose, data from subjects with an age ±5 years of the patient’s age. Thus, for example, for a patient with an average mean age of DO patients, 41 years (see Table 1), data from 39 healthy controls would be used. As indicated in Equation (1), the BCI uses a combination of stance and gait trunk sway and trial duration measures which can be either excessively large or overly small (with long trial durations). It has been validated in a number of patient groups, including those with structural and functional disorders as well as those mimicking balance problems [14,27,28,29,37].

To quantify patients’ use of visual and somatosensory inputs to control balance during 2-legged stance, we computed the following control ratios (*CR*), respectively:

(2) $${CR}_{vs}=\left(\frac{\left(v_{s2ecf}-v_{s2eof}\right)+\left(v_{s2ec}-v_{s2eo}\right)}{\left({v_{s2eo}+v_{s2ec}+v}_{s2eof}+v_{s2ecf}\right)}\right)\;*\;100$$

(3) $${CR}_{ss}=\left(\frac{\left(v_{s2ecf}-v_{s2ec}\right)+\left(v_{s2eof}-v_{s2eo}\right)}{\left({v_{s2eo}+v_{s2ec}+v}_{s2eof}+v_{s2ecf}\right)}\right)\;*\;100$$

where *v* stands for peak-to-peak pitch velocity for the particular task as defined above. These ratios were compared with those of the age-matched controls in a similar manner as for the BCI.

### 2.5. Intervention: Balance Training with Vibro-Tactile Feedback of Trunk Sway

Vibro-tactile feedback (VTfb) of trunk sway was provided to study participants using an add-on device of Swaystar called Balance Freedom™ and approved by the Swiss Agency for Therapeutic Products (Swissmedic Reference Number: 2008-MD-0011) for use on patients. This feedback system consists of 8 vibrators positioned at 45-degree intervals around a circular headband, thereby providing directionally specific sway information (see Figure 1 in Allum et al. [27]). For example, if a sway threshold for forward pitch was exceeded, the vibrator in the middle of the forehead was activated. Likewise, for backward movement exceeding threshold, the vibrator at the back of the head came on. Left and right supra-threshold sway caused the vibrators over the left and right ears, respectively, to be activated. For sway in the diagonal directions, forward and left, for example, the vibrator between the forehead and left ear was activated when its threshold was exceeded. Thus, when the head was aligned in the straight-ahead position, the vibro-tactile feedback (VTfb) provided by the vibrators was aligned with the axes of the SwayStar gyroscopes mounted at lumbar 5. The controller for the vibrators was connected directly with the SwayStar™ unit. Task-specific thresholds for trunk sway angles were set in the controller based on pitch and roll measures obtained from the first (baseline) assessment and readjusted based on the assessment after the first week of training. The threshold VTfb amplitudes were calculated as 40% of the 90% ranges of pitch and roll sway angle. We used 40% of the 90% ranges based on a previous assessment of the average range of sway reductions achieved by healthy elderly and young normal subjects (35). Thus, the threshold ranges were set at 80% (40% for each side) of the 90% ranges. To determine the 90% ranges, the total peak-to-peak range of each trunk sway variable was determined over the trial duration for a task, and this range was split into 40 bins. Samples were then sorted into the appropriate bins to build a histogram of the samples. The range from the lower 5% level to the upper 95% level of the histogram defined the 90% range.

### 2.6. Procedure

Over a period of 3 weeks, all participants performed assessment and training sequences based on the sequence of 14 gait and stance tasks described above. The assessment sequence comprised all 14 tasks. However, the training sequence had only 11 tasks, as the 3 one-legged stance tests were not used for training as most subjects could only perform these tasks for a few seconds. During training, each of the 11 tasks was performed 4 times. The order of assessments and training sessions was as follows: First, an assessment sequence with the 14 stance and gait tasks was performed for use as the baseline assessment. The thresholds for training performed over the following 7 days were calculated from this baseline assessment. During these 7 days, 2 training sessions were scheduled at least 2 days apart, for example, on Monday and Wednesday. Shortly after the second training session, that is, after a rest of 5 to 10 min to avoid fatigue, a second assessment sequence was performed in order to be able to reset the thresholds based on any changes in balance control that occurred in the previous 7 days. In the following 7 days, the 2 training sessions and 1 assessment session were repeated. The VTfb device was not worn as a placebo during the assessment sessions. Training sessions lasted approximately 45 min and assessment sessions lasted 20 min. Assessments and training were always performed without shoes to avoid balance variations due to shoe types. To avoid diurnal differences in fatigue levels affecting the results, assessments and training sessions were almost always performed at the same preferred time of the day for each patient.

### 2.7. Rationale for Total Duration of VTfb Training and Assessment Sessions

The current study was designed to determine the magnitudes of improvements in balance control elicited by 2 weeks of VTfb training and observable as a subsequent carry-over effect in the assessment sequences. In prior studies, Rust et al. [38] and Allum et al. [27] noted that a major 20% improvement in the balance control of multiple sclerosis (MS) patients occurred in the first week of VTfb training. Thereafter, if the training was continued for another 3 weeks, there was a slight further improvement over time with the carry-over effect lasting some 3–4 months [27]. Therefore, for this initial study on PPPD patients, the total duration of the VTfb training and assessment sessions was limited to 2 weeks, as we assumed that a similar pattern of improvements as observed with the patients of our previous studies would be seen with the PPPD patients.

### 2.8. Patient Clinical Characteristics

Within each group of patients (QBD and DO), we noted (see Table 1) whether dizziness was constantly present or not, the presence or absence of a structural vestibular deficit, the presence of phobic postural vertigo defined according to the criteria of Querner et al. [13] and Brandt et al. [33], and the presence or absence of lower leg polyneuropathy. For all of the 23 patients, dizziness was constant (occurring on 15 days or more each month, according to the accepted criterion [1]), and therefore, they could be diagnosed as having persistent postural-perceptual dizziness (PPPD). One QBD patient had periodic dizziness and benign postural positioning nystagmus. As indicated in Table 1, 5 of the 12 QBD patents and 3 of the 11 DO patients had a chronic structural peripheral vestibular loss as determined by caloric tests [31], that is, a canal paresis value greater than 30% [31], or by a test of the lateral gain of the vHIT being less than 0.7. or by the presence of benign proximal positioning nystagmus (BPPN). In all these patients, except the BPPN patient, the vestibular loss was assumed to be due to vestibular neuritis based on the clinical signs on acute clinical manifestation and subsequent caloric and video head impulse tests (vHIT) [39]. Seven of the QBD patients and two of the DO patients had phobic postural vertigo defined according to the criteria of Querner et al. [13] and Brandt et al. [33]. That is, the patients had pathological balance control, as scored with the BCI, and performed more difficult balance tasks with better scores than easier balance tasks of the same type, e.g., walking 8 tandem steps on foam compared to walking on a normal, firm support surface. Two of the QBD patients and none of the DO patients had a lower-leg polyneuropathy. 

### 2.9. Statistical Analysis

A repeated-measures analysis of variance was performed with IBM SPSS Statistics to estimate possible differences between the QBD and DO groups’ mean values prior to the onset of VTfb training, compared to after 1 week and after 2 weeks of training. Post hoc paired *t*-tests were calculated between mean values at each test time across both groups as well as separately for each group. The level of significance was set at 0.05 before taking into account Bonferroni corrections, implying for the 3 comparisons over time that the level of significance should be set at alpha equals 0.016. As this level was reached in the majority of tests of BCI values, the 2-sided *p* values listed in the figures and Table 2 were left uncorrected. In our study, we depended on the BCI (Equation (1)), which is an additive score of sway metrics developed using stepwise discriminant analysis of sway metrics from stance and gait tests recorded from vestibular deficit patients and normal controls [29], such that the sway values entered into the discriminant function have the highest significance once correlations between the to-be-entered variables and already-entered variables are taken into account. Having established that this BCI score is significant post- versus pre-treatment, we then examined, as listed in Figure 2 and Table 2, the possible contributions of the entered sway metrics to these BCI scores, as well as the differences pre- and post-treatment of the remaining non-entered test sway metrics.

## 3. Results

The feedback variable signalled to the patients was the amplitude of trunk sway in the form of a vibrator activation applied at the head when the sway exceeded 0.4 times the previously measured 90% range of pitch or roll sway. Assuming that these were the primary variables controlled by the subjects, as we specifically asked them to avoid activating the vibrators, we first examined the effect of the feedback on these ranges (Table 2). Based on previous research [28,40], we expected that the subjects with pathological balance control would show different improvements in balance control with VTfb training than those with normal balance control. Thus, for the purposes of examining the global balance (BCI) and dizziness (DHI) indexes, we separated our PPPD patients into two groups, those with and without pathological balance control, as measured with the BCI value (see Table 1 and Figure 3, Figure 4 and Figure 5)—an index that contains both pitch and roll sway angle and angular velocity terms [29,30]. Prior to examining the improvements in the mean 90% amplitudes of trunk sway, the BCI and DHI values with VTfb balance training for the separate DO and QBD populations, we established, with repeated measures of ANOVAs, that a highly significant time effect was present in the BCI and DHI scores (F = 34.2 and 18.5, respectively, *p* < 0.001) for the combined data of both groups.

### 3.1. Effects of Vibro-Tactile Feedback (VTfb) Balance Training on Trunk Sway

**Figure 1:**
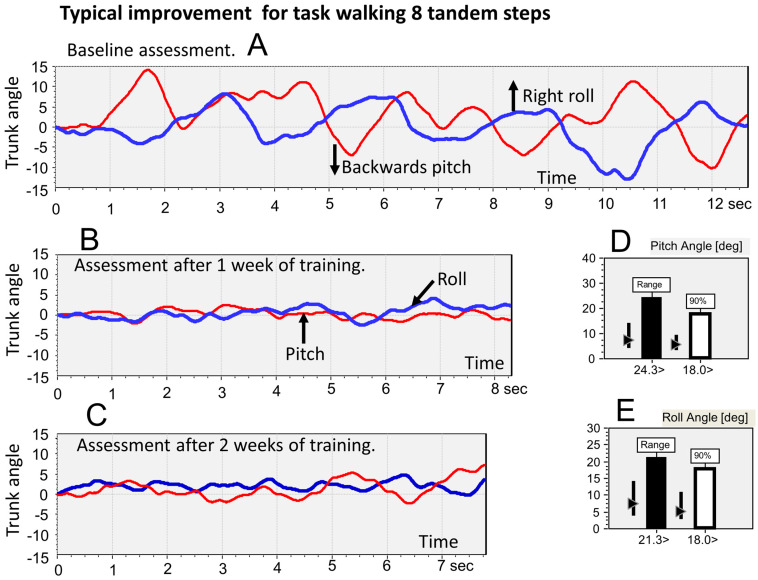
Example of the reduction in pitch and roll lower-trunk sway amplitudes for tandem gait trials of a dizziness only (DO) patient, compared to baseline (upper traces, (**A**)). Traces recorded after 1 week of training with vibro-tactile feedback (VTfb) of lower-trunk pitch and roll sway angles are shown in (**B**) (middle traces), and after 2 weeks of training in (**C**) (lower traces). Note how most improvement (reduction in amplitude) in trunk sway occurs during the first week of training as shown in (**B**) compared to the sway after 2 weeks of training shown in (**C**). The inserts (**D**,**E**) compare the peak-to-peak (Range) and 90% values for the data shown in (**A**) with normal reference values of controls. The vertical bars next to the columns in (**D**,**E**) mark the 90% range of controls, and the triangles on the vertical bars mark the median values of controls. The values for the data of (**A**) are listed below the columns in (**D**,**E**). The range and 90% values are 24.3 and 18.0 in (**D**), and 21.3 and 18.0 in (**E**). VTfb thresholds computed from the baseline assessment (**A**) were ±7.2 deg (18.0 × 0.4) for pitch and roll. After 1 week of training, these thresholds were reduced to ±1.4 deg for pitch and ±1.8 for roll.

**Figure 2:**
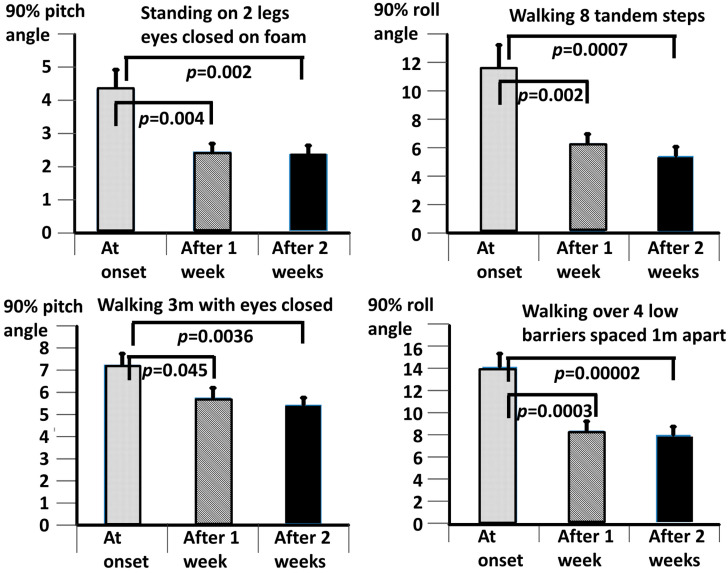
Changes in 90% ranges of trunk sway angles after 1 and 2 weeks of VTfb training for 2 eyes closed trials (standing eyes closed on foam and walking 3 m eyes closed) and 2 difficult gait tasks (walking tandem steps and walking over low barriers). Combined (DO and QBD) population values are shown in each set of column plots. The mean population value is represented by the height of the column in each column plot, and the standard error of the mean (sem) is represented by the vertical bar on the top of each column. Significant differences between mean onset (baseline) values and mean values after 1 and 2 weeks of training are marked by horizontal bars and the respective *t*-test probability (*p*) values. There were no significant differences between mean values after 1 and 2 weeks of VTfb training.

Figure 1 illustrates a typical finding for gait trials (see Table 2) across our PPPD populations, namely, that for the task of tandem walking on a normal floor, roll and pitch trunk movements were greater in amplitude during the baseline assessment than those of the age-matched healthy control subjects. Often (9 of 23 cases—see Table 1), as was the case for the patient in Figure 1, the trunk sway was less when performing the same task on a foam surface, thereby fulfilling the criteria of phobic postural vertigo according to Querner et al. [13]. After 1 week of VTfb training, the trunk sway amplitudes as noted in Figure 1D,E and Figure 2 (upper right)—compare the vertical bars in Figure 1D,E with the columns in Figure 2—decreased to be well within the normal range of the age-matched controls. Following a further week of VTfb training, the improvement was maintained, but not significantly increased (compare middle traces with lower traces in Figure 1B,C, respectively).

The changes in balance control, seen in Figure 1, were replicated in the QBD and DO population mean values as listed in Table 2. Table 2 lists the mean percentage of improvements and significance thereof for each of the 11 training tasks. Several features of Table 2 are noteworthy. Firstly, all variables showed an improvement (positive values). However, for several variables, the change was not significant. Secondly, more pitch than roll values were significant. Thirdly, taking into account the number of stance and gait tasks, proportionally more gait than stance improvements were significant. 

The differences seen in Table 2 translated into significant changes with VTfb training across the combined QBD and DO populations. In Figure 2, in addition to the task of walking tandem steps, the population values for three other tasks included in the BCI measure are also shown. For each task in Figure 2, the trunk sway amplitudes at 2 weeks were significantly less than the onset measures (*p* ≤ 0.004). In contrast, the significance for each task between the onset and 1-week values was always less (*p* ≤ 0.045). Furthermore, there was no difference between the 1 and 2-week values (*p*, 2-sided, ≥0.05).

### 3.2. Effects of Vibro-Tactile Feedback (VTfb) Balance Training on Balance Control and Dizziness Handicap Indexes

**Figure 3:**
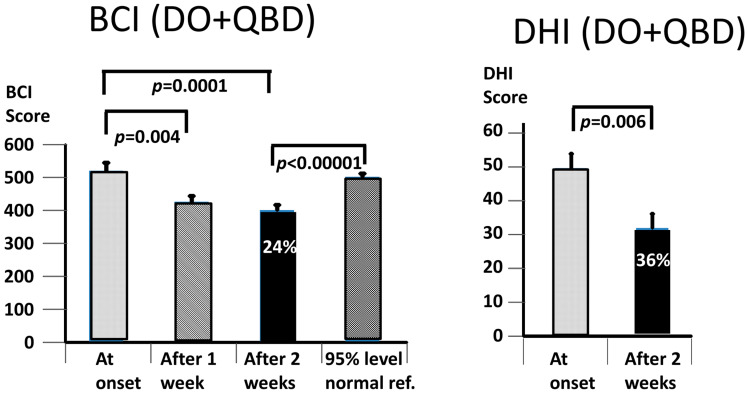
Improvement of Balance Control Index (BCI) values and Dizziness Handicap Inventory (DHI) scores for combined DO and QBD subject data. The percentage values on the column plots for 2 weeks are the percentage reduction achieved with respect to baseline values. The layout of the figure is identical to that of Figure 2 except that a column has been added in the left column plot displaying the mean of the 95% age-matched reference value for the BCI.

When the BCI values were combined for both PPPD populations (see Figure 3), there was a significant decrease after 1 week of training (*p* = 0.004), with a further decrease after 2 weeks (*p* = 0.0001). As indicated in Figure 3, the average improvement after 2 weeks was 24%. This improvement was to a significantly lower BCI value than the average upper 95% BCI amplitude of the age-matched controls (*p* < 0.00001, see Figure 3). The improvement in the DHI values over 2 weeks was 36%, but less significant (*p* = 0.006) than the change in the BCI values—see Figure 3.

**Figure 4:**
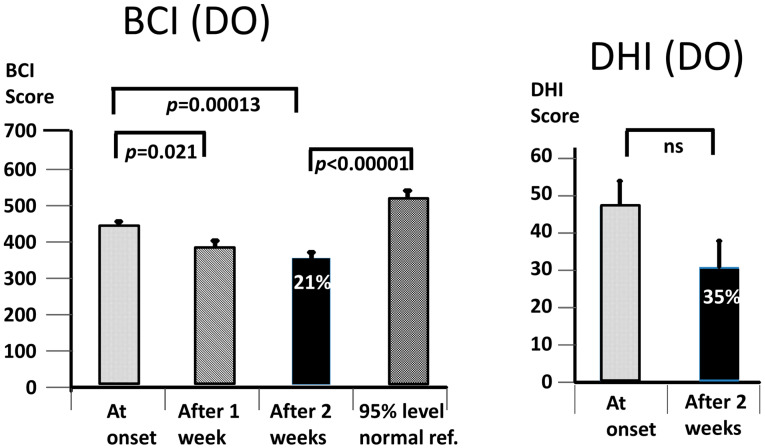
Improvement of Balance Control Index (BCI) values and Dizziness Handicap Inventory (DHI) scores for DO subject data only. The layout of the figure is identical to that of Figure 3. ns, stands for not significant.

**Figure 5:**
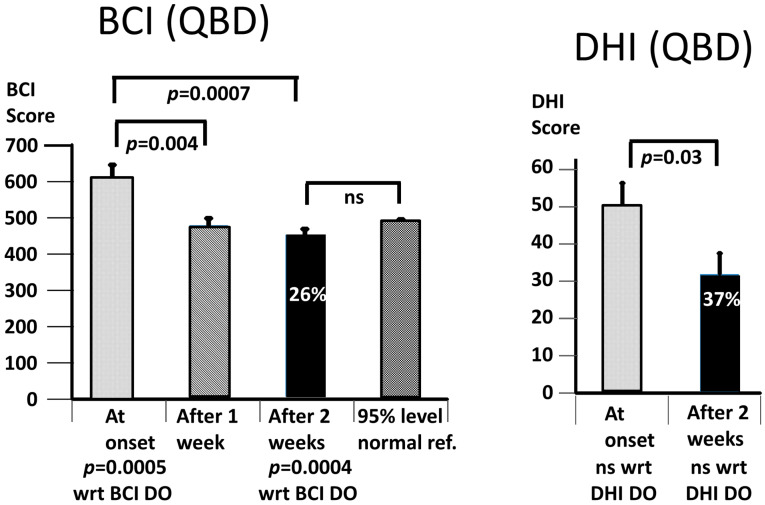
Improvement of Balance Control Index (BCI) values and Dizziness Handicap Inventory (DHI) scores for QBD subject data only. The layout of the figure is identical to that of Figure 3. ns, stands for not significant.

As expected, the BCI scores at the onset were greater for the QBD group (*p* = 0.0005) than for the DO group (Figure 5). Likewise, the BCI score at 2 weeks (*p* = 0.0004) was also greater—see Figure 5. Interestingly, the DHI scores were not different between the two groups, neither at onset nor after 2 weeks of training—see Figure 4 and Figure 5. Furthermore, the percentage changes in the DHI values were very similar (35 and 37% for the DO and QBD groups, respectively). The change was, however, only significant for the QBD group. Furthermore, the change in the mean DHI scores (19) for the QBD group was very similar to the minimum clinically important difference (18) for those with vestibular deficits [34]. In contrast, the 21% change in the BCI value for the DO group between the onset and 2 weeks was highly significant (*p* = 0.00013), as was the larger 26% change for the QBD group (*p* = 0.0007). As the onset BCI values for the DO subjects were always lower than the 95% value of the age-matched healthy controls by group entry definition, it was not surprising that the BCI value at 2 weeks was statistically less than the average 95% normal control reference value (*p* < 0.00001). For the QBD group, there was no statistical difference between the 2-week value and the average 95% normal reference value.

## 4. Discussion

Our working hypothesis that both our primary measures, the BCI and the DHI, would be improved by the vibro-tactile intervention proved to be partially false, as only the BCI was significantly improved. The intervention clearly improved trunk sway during the stance and gait tasks for both the DO and QBD patients, but did not significantly improve the perceived dizziness handicap as measured with the DHI for the DO patients. In the QBD patients, there was only a weakly significant improvement in the DHI scores. We hypothesised that the improved functional stance and gait comorbidities of the patients would lead to decreased sensations of dizziness as a result of the sensory reweighting of the central motor commands. There was a significant difference, as measured with the BCI score at 2 weeks, in the effect of the vibro-tactile feedback training on trunk sway between the two groups. The BCI scores of the DO group were lower (see Figure 4 and Figure 5). However, this was not the case for the DHI scores. There was no difference in the DHI scores between the two groups at 2 weeks (see Figure 5). That is, the DHI scores in the DO patients were not lower than in the QBD group. Therefore, these results suggest that vibro-tactile feedback improved the functional stance and gait comorbidities in patients with PPPD more than the dizziness itself. That is, these two effects caused by the intervention appear not to be correlated with one another. Because the stance tests were generally less influenced by the intervention (see Table 2), future studies should concentrate on these tests, possibly using a different intervention from that of this study, but retaining the vibro-tactile feedback intervention for the gait tests.

Using standard procedures for treating PPPD [41], that is, vestibular rehabilitation (VR) combined with cognitive behavioural therapy (CBT), more modest improvements (5%) in the balance control (BCI values) were achieved for the DO and QBD groups [23]. Possibly, this was because CBT was a major part of the therapy, rather than VTfb training as in the current study. On the other hand, CBT yielded improvement in a number of psychometric measures, for example, obsession, compulsiveness, and phobic anxiety in the DO patients, but only a reduction in phobic anxiety for the QBD patients [12,23]. When viewed from the combined perspective of the Schmid et al. [12,23] studies and the current study, we would suggest, as others [42], that the gait exercises performed during VR could be more effective if performed with accompanying VTfb. Thus, it is an open question whether balance control would improve more when CBT is combined with VTfb balance training.

Because we had two patient populations, one with balance control deficits (QBD) with respect to the age-matched controls, and the other with dizziness alone (DO), both of whom improved their balance control with training, we would suggest that because the afferent feedback gains of both groups improved with vibro-tactile feedback (VTfb) training, this possibly suggests that similar central nervous processes were used for this purpose. However, it is a shortcoming of the current study that we did not test whether the actual perception of trunk sway was different between the two groups. Thus, it is possible that the perceived sway was greater than the actual sway in the QBD group, as seen in studies investigating the effect of postural threat on perceived trunk sway [26]. Cleworth et al. [26], in their studies, observed no increase in the lateral trunk sway with support-surface roll rotations at a height of 3.2 m with respect to 1.1 m. However, a significant increase in the perceived trunk sway was observed. The relationship between the perceived and actual sway was recently investigated in PPPD patients by San Pedro Murillo et al. [15] for the task of standing on two legs with eyes closed on a firm surface. By showing a video of the patient’s body movements and explaining the centre of foot pressure traces in relation to PPPD, the patients could reduce their perceived sway. However, the amplitude of the observed sway did not change. Our findings, coupled with those cited above, suggest that there are two neural processes underlying PPPD, perception and feedback control of body sway, which may be improved by artificial feedback of trunk sway or cognitive behavioural therapy-like explanations, or may be worsened by postural threat. It remains to be explored why, as indicated in Table 2, the improvements in perceived sway and sway motor signals are not similar across the stance and gait tasks, and whether it is possible to measure perceived sway during gait tasks without introducing a multi-tasking effect on balance control.

The question arises over which sensory channels we were providing artificial feedback of trunk sway. Our intention was to provide vibro-tactile information on trunk sway angle thresholds being exceeded in one of eight directions by switching on a vibrator active at 250 Hz at the head in the same direction. There were a number of reasons for this choice of 250 Hz. Firstly, the 250 Hz vibration occurred within the range of vibration frequencies which excite skin receptors [43]; secondly, the 250 Hz vibration is outside of the frequency for exciting the group Ia muscle stretch receptors. Usually, 80 Hz vibration is used for this purpose [44,45]. Thirdly, even if 250 Hz vibration applied at the head at the level of the forehead excited the neck muscle Ia receptors, the induced body sway would presumably be much smaller than with direct 80 Hz vibration at the muscle [45]. Hence, given its small displacement amplitude and high frequency, we assumed that the vibrators activated almost exclusively cutaneous receptors. It should be stated that this conclusion is probably not valid when the vibration is applied at the waist and could excite muscle receptors in the abdominal and paraspinal muscles, and thereby induce trunk sway [45,46,47]. 250 Hz vibration applied to the head can, however, excite otolith receptors via bone conduction [48,49]. Thus, it remains to be investigated whether the vibrations we applied at different locations around the head led to a coding of vibration-induced perceived tilt and nystagmus via utricle receptors. Utricle receptors code the direction of head and trunk tilt when the trunk and head are tilted together, as is the case for movements below 3 Hz for quiet stance [50].

The question also arises whether training alone, without VTfb, would have achieved an equally significant effect on the balance scores. Previous work on multiple sclerosis (MS) patients [27], Parkinson’s disease patients [51], and the healthy elderly [40,42] indicate that the balance scores improved more as a carry-over effect of training with VTfb than without. Based on these results, the subsidiary question becomes one of the number of sessions required to achieve a significant improvement with respect to the baseline test values, and how much this improvement increases with additional training. In our previous work with MS patients [27] and in the current study (see Figure 3, Figure 4 and Figure 5), we noted a major effect in the first week of training, followed by small further improvements in the subsequent weeks, such that the improvement between the first and second week was not significant. Likewise, for those trial patients for whom we provided a third week of VTfb training, there was no significant improvement between the first and third week (preliminary unpublished data). Therefore, we would argue that 2 weeks of training is sufficient to achieve a significant improvement in balance control.

In this study, we have used the carry-over effect of vibro-tactile feedback (VTfb) training of trunk sway to reduce the observed sway and DHI scores by about 25% in PPPD patients. During the training sessions, when on-line feedback was provided in the form of an activation of a vibrator when the pre-set trunk (L5) sway threshold was reached, the subjects’ instruction was to move in the opposite direction to the activated vibrator in order to switch it off (see Methods Section 2). The underlying logic for this approach was based on our studies with MS patients [27], which demonstrated that the differences between the on-line and carry-over induced effects were small, and the carry-over effects were still present 1–2 months after the training had ceased. That is, we assumed that the sensory gain adjustments induced during training were maintained when vibro-tactile feedback was not available. The advantages of this approach are that the PPPD patients do not have to wear the feedback device all the time to obtain an improvement in balance control, and do not have to adjust the feedback device thresholds to their current balance task, e.g., standing or walking. It remains to be investigated in future studies whether the small difference in between on-line and carry-over effects observed for MS patients [27] is also the case for PPPD patients.

As far as we are aware, this is the first study to document an effect of vibro-tactile feedback on trunk sway angle for PPPD patients. Other investigators have used different types of feedback, for example, combined trunk sway angle and angular velocity [52], or stepwise increases in the vibration pulse rate dependent on the head sway angle [53] to examine whether sway was improved in their vestibular-loss patient groups. Thus, future studies should also investigate whether improved carry-over effects are also observed for PPPD patients with these and other types of trunk sway feedback devices.

## 5. Conclusions

These results show that providing VTfb of trunk sway to PPPD subjects yields a significant improvement in balance control, as measured with trunk sway during stance tasks and a more significant improvement in gait tasks. The benefit is greater for the QBD group of PPPD patients with initially pathological trunk sway than for the dizziness only (DO) group with initially normal trunk sway. For the QBD PPPD patients, the intervention had a much weaker effect on the influence of dizziness on daily living as measured with the DHI questionnaire scores. The effect on the DHI scores was not significant in the DO PPPD patients. 

These results suggest that the physiologic processes underlying sensorimotor control of stance and gait and the perception of movement do not operate in lockstep, as has shown to be the case in normal individuals [26], nor are correlated with one another as has been shown previously in PPPD patients [23]. In PPPD, this natural dissociation seems to be enlarged, which has substantial implications for pathophysiologic models of PPPD and future developments in treatment.

## Figures and Tables

**Table 1:** Demographic data of quantified balance deficit (QBD) and dizziness only (DO) subjects.

	Quantified Balance Disorders (QBD) Group	Dizziness Only (DO) Group
N	12	11
Age mean ± SD ►	53.4 ± 12.7 years	40.6 ± 14.1 years
Gender M/F	6/6	7/4
Pathological BCI ●	12/12	0/11
Pathological visual control during 2-legged stance Ѧ	7/12	2/11
Pathological somatosensory control Ѧ	6/12	2/11
Dizziness constant (fitting definition of PPPDφ) or occurring in episodes	12 constant/1 in episodes	11 constant/0 in episodes
Phobic postural vertigo *	7/12	2/11
Deficits in VOR# tests	5/12 ▲	3/11

►—Difference in mean age significant (*p* = 0.03). ●—BCI stands for Balance Control Index, which is a combination of measurement values from stance and gait tests [29,30]—see Section 2 (Methods). PPPDφ (persistent postural-perceptual dizziness) is defined according to the criteria of Staab et al. [1] and requires that dizziness be present for 15 of every consecutive 30 days. Ѧ—Visual control ratio for 2-legged stance significantly larger for QBD than DO group, *p* = 0.04. Somatosensory control ratio, no significant difference between groups. #VOR stands for vestibular ocular reflex. To be defined as a peripheral vestibular deficit (PVD) patient, the caloric paresis value had to be greater than 30%, or the lateral gain in vHIT had to be less than 0.7. All PVD patients had either a chronic uncompensated or decompensated unilateral PVD. The VOR responses of the decompensated patients were similar to those with bilateral peripheral vestibular deficit; gains were lower than normal in both directions of yaw rotation. *—The presence of phobic postural vertigo is based on the criteria of Brandt et al. [33] supported by the findings of Querner et al. [13]. ▲—1 with additionally benign proximal positioning nystagmus.

**Table 2:** Percentage differences and significance (uncorrected for multiple comparisons) of the effect of VTfb balance training on pitch and roll trunk sway 90% angle ranges for trained tests. The differences were calculated between test trunk sway at baseline, prior to training onset, and after 2 weeks of training.

Test	QBD	DO	QBD	DO
	pitch	pitch	roll	roll
s2eo	ns 24.9	ns 21.1	ns 13.2	ns 42.6
s2eof	ns 13.5	**0.014** **43.4**	ns 21.1	ns 35.2
s2ec	ns 16.1	ns 31.3	ns 37.4	ns 30.9
s2ecf	ns 34.6	**0.025** **60.6**	ns 19.4	**0.03** **48.7**
w8tan	**0.016** **54.0**	**0.013** **53.3**	**0.025** **50.2**	**0.007** **56.8**
w8tanf	**0.006** **54.1**	ns 37.5	**0.021** **52.0**	**0.005** **49.9**
w3mhr	**0.03** **23.5**	**0.006** **34.6**	ns 13.1	ns 22.2
w3mhp	**0.04** **27.1**	ns 17.8	ns 26.6	ns 15.8
w3mec	**0.036** **23.0**	**0.05** **26.8**	ns 56.0	ns 26.1
stairs	**0.049** **23.7**	**0.031** **27.6**	ns 22.5	**0.039** **23.3**
barriers	**0.006** **37.0**	**0.0014** **29.3**	**0.0005** **47.4**	**0.0021** **41.7**
N of 11 significant	**7**	**7**	**3**	**5**

The significance is listed as the probability of a significant 2-sided *t*-test on the difference between the means at baseline and after 2 weeks of training. Significant results are highlighted with bold text. ns stands for not significant. Note that the difference in means is always positive (an improvement, that is, less sway). Measures from the following tests are used in the BCI computation—see methods section: s2ecf, w8tan, w3hr, w3hp, w3ec, and stairs. The following abbreviations are used: s2 for standing on 2 legs, ec for eyes closed, f for foam, w8tan for walking 8 tandem steps, w3 for walking 3 m, hr for head rotating, and hp for head pitching. The number (N) of significant improvements in pitch or roll sway is listed in the last row of the table.

## Data Availability

Data supporting the results of this study may be requested from thecorresponding author.

## References

[1] Staab J.P., Eckhardt-Henn A., Horii A., Jacob R., Strupp M., Brandt T., Bronstein A. (2017). Diagnostic criteria for persistent postural-perceptual dizziness (PPPD). Consensus document of the Committee for the Classification of Vestibular Disorders of the Bárány Society. J. Vestib. Res..

[2] Brandt T. (1996). Phobic postural vertigo. Neurology.

[3] Bronstein A.M. (1995). The visual vertigo syndrome. Acta Otolaryngol..

[4] Jacob R.G., Woody S.R., Clark D.B., Lilienfeld S.O., Hirsch B.E., Kucera G.D., Furman J.M., Durrant J.D. (1993). Discomfort with space and motion: A possible marker of vestibular dysfunction assessed by the situational characteristics questionnaire. J. Psychoathol. Behav. Assess..

[5] Ruckenstein M.J., Staab J.P. (2009). Chronic subjective dizziness. Otolaryngol. Clin. N. Am..

[6] Popkirov S., Staab J.P., Stone J. (2018). Persistent postural-perceptual dizzi-ness (PPPD): A common, characteristic and treatable cause of chronic dizziness. Pract. Neurol..

[7] Nazareth I., Yardley L., Owen N., Luxon L. (1999). Outcome of symptoms of dizziness in a general practice community sample. Fam. Pract..

[8] Kim H.J., Lee J.O., Choi J.Y., Kim J.S. (2020). Etiologic distribution of dizziness and vertigo in a referral-based dizziness clinic in South Korea. J. Neurol..

[9] Xue H., Chong Y., Jiang Z.D., Liu Z.L., Ding L., Yang S.L., Wang L., Xiang W.P. (2018). Etiological analysis on patients with vertigo or dizziness. Zhonghua Yi Xue Za Zhi.

[10] Castro P., Bancroft M.J., Arshad Q., Kaski D. (2022). Persistent Postural-Perceptual Dizziness (PPPD) from Brain-Imaging to Behaviour and Perception. Brain Sci..

[11] Dieterich M., Staab J. (2017). Functional dizziness: From phobic postural vertigo and chronic subjective dizziness to persistent postural-perceptual dizziness. Curr. Opin. Neurol..

[12] Schmid D.A., Allum J.H.J., Sleptsova M., Gross S., Gaab J., Welge-Lüssen A., Schaefert R., Langewitz W. (2018). Effects of a program of cognitive-behavioural group therapy, vestibular rehabilitation, and psychoeducational explanations on patients with dizziness and no quantified balance deficit, compared to patients with dizziness and a quantified balance deficit. J. Psychosom. Res..

[13] Querner V., Krafczyk S., Dieterich M., Brandt T. (2000). Patients with somatoform phobic postural vertigo: The more difficult the balance task, the better the balance performance. Neurosci. Lett..

[14] Vonk J., Horlings C.G.C., Allum J.H.J. (2010). Differentiating malingering balance disorder patients from healthy controls, compensated unilateral vestibular loss, and whiplash patients using stance and gait posturography. Audiol. Neurootol..

[15] San Pedro Murillo E., Bancroft M.J., Koohi N., Castro P., Kaski D. (2022). Postural misperception: A biomarker for persistent postural perceptual dizziness. J. Neurol. Neurosurg. Psychiatr..

[16] Lempert T., Olesen J., Furman J., Waterston J., Seemungal B., Carey J., Bisdorff A., Versino M., Evers S., Newman-Toker D. (2012). Vestibular migraine: Diagnostic criteria. J. Vestib. Res..

[17] Tan C.J., Shufelt T., Behan E., Chantara J., Koomsri C., Gordon A.J., Chaiyakunapruk N., Dhippayom T. (2023). Comparative effectiveness of psychosocial interventions in adults with harmful use of alcohol: A systematic review and network meta-analysis. Addiction.

[18] Schmid G., Henningsen P., Dieterich M., Sattel H., Lahmann C. (2011). Psychotherapy in dizziness: A systematic review. J. Neurol. Neurosurg. Psychiatr..

[19] Edelman S., Mahoney A.E.J., Cremer P. (2012). Cognitive behavior therapy for chronic subjective dizziness: A randomized, controlled trial. Am. J. Otolaryngol..

[20] Holmberg J., Karlberg M., Harlacher U., Magnusson M. (2007). One-year follow-up of cognitive behavioral therapy for phobic postural vertigo. J. Neurol..

[21] Holmberg J., Karlberg M., Harlacher U., Rivano Fischer M., Magnusson M. (2006). Treatment of phobic postural vertigo: A controlled study of cognitive-behavioral therapy and self-controlled desensitization. J. Neurol..

[22] Mahoney A.E., Edelman S., Cremer P.D. (2013). Cognitive behavior therapy for chronic subjective dizziness: Longer-term gains and predictors of disability. Am. J. Otolaryngol..

[23] Schmid D.A., Allum J.H., Sleptsova M., Welge-Lüssen A., Schaefert R., Meinlschmidt G., Langewitz W. (2020). Relation of anxiety and other psychometric measures, balance deficits, impaired quality of life, and perceptive state of health to dizziness handicap inventory scores for patients with dizziness. Health Qual. Life Outcomes.

[24] Derogatis L.R., Melisaratos N. (1983). The Brief Symptom Inventory: An introductory report. Psychol. Med..

[25] Naranjo E.N., Cleworth T.W., Allum J.H.J., Inglis J.T., Lea J., Westerberg B.D., Carpenter M.G. (2016). Vestibulo-spinal and vestibulo-ocular reflexes are modulated when standing with increased postural threat. J. Neurophysiol..

[26] Cleworth T.W., Adkin A.L., Allum J.H.J., Inglis J.T., Chua R., Carpenter M.G. (2019). Postural threat modulates perceptions of balance related movement during support surface rotations. Neuroscience.

[27] Allum J.H.J., Rust H.M., Lutz N., Schouenborg C., Fischer-Barnicol B., Haller V., Derfuss T., Kuhle J., Yaldizli Ö. (2021). Characteristics of improvements in balance control using vibro-tactile biofeedback of trunk sway for multiple sclerosis patients. J. Neurol. Sci..

[28] Honegger F., Hillebrandt I.M.A., van den Elzen N.G.A., Tang K.-S., Allum J.H.J. (2013). The effect of prosthetic feedback on the strategies and synergies used by vestibular loss subjects to control stance. J. Neuroeng. Rehabil..

[29] Allum J.H., Adkin A.L. (2003). Improvements in trunk sway observed for stance and gait tasks during recovery from an acute unilateral peripheral vestibular deficit. Audiol. Neurootol..

[30] Hegeman J., Shapkova E.Y., Honegger F., Allum J.H.J. (2007). Effect of age and height on trunk sway during stance and gait. J. Vestib. Res..

[31] Allum J.H., Ura M., Honegger F., Pfaltz C.R. (1991). Classification of peripheral and central (pontine infarction) vestibular deficits. Selection of a neuro-otological test battery using discriminant analysis. Acta Otolaryngol..

[32] Callaghan B., Price R., Feldman E. (2015). Distal Symmetric Polyneuropathy: A Review. JAMA.

[33] Brandt T., Dieterich M., Strupp M. (2013). Somatoform Vertigo and Dizziness Syndromes. Vertigo and Dizziness: Common Complaints.

[34] Jacobson G.P., Newman C.W. (1990). The development of the Dizziness Handicap Inventory. Arch. Otolaryngol. Head. Neck Surg..

[35] Volz-Sidiropoulou E., Takahama J., Gauggel S., Westhofen M. (2010). Das “Dizziness Handicap Inventory”: Erste psychometrische Kennwerte einer Deutschen Version. Laryngorhinootologie.

[36] Nola G., Mostardini C., Salvi C., Ercolani A.P., Ralli G. (2010). Validity of Italian adaptation of the Dizziness Handicap Inventory (DHI) and evaluation of the quality of life in patients with acute dizziness. Acta Otorhinolaryngol. Ital..

[37] Allum J.H., Carpenter M.G., Adkin A.L. (2001). Balance control analysis as a method for screening and identifying balance deficits. Ann. N. Y. Acad. Sci..

[38] Rust H.M., Lutz N., Zumbrunnen V., Imhof M., Yaldizli Ö., Haller V., Allum J.H. (2020). Benefits of short-term training with vibrotactile biofeedback of trunk sway on balance control in multiple sclerosis. Phys. Med. Rehabil. Res..

[39] MacDougall H.G., Weber K.P., McGarvie L.A., Halmagyi G.M., Curthoys I.S. (2009). The video head impulse test: Diagnostic accuracy in peripheral vestibulopathy. Neurology.

[40] Davis J.R., Carpenter M.G., Tschanz R., Meyes S., Debrunner D., Burger J., Allum J.H. (2010). Trunk sway reductions in young and older adults using multi-modal biofeedback. Gait Posture.

[41] Trinidade A., Goebel J.A. (2018). Persistent Postural-Perceptual Dizziness—A Systematic Review of the Literature for the Balance Specialist. Otol. Neurotol..

[42] Bao T., Carender W.J., Kinnaird C., Barone V.J., Peethambaran G., Whitney S.L., Kabeto M., Seidler R.D., Sienko K.H. (2018). Effects of long-term balance training with vibrotactile sensory augmentation among community-dwelling healthy older adults: A randomized preliminary study. J. Neuroeng. Rehabil..

[43] Vedel J.P., Roll J.P. (1982). Response to pressure and vibration of slowly adapting cutaneous mechanoreceptors in the human foot. Neurosci. Lett..

[44] Roll J.P., Vedel J.P. (1982). Kinaesthetic role of muscle afferents in man, studied by tendon vibration and microneurography. Exp. Brain Res..

[45] Courtine G., De Nunzio A.M., Schmid M., Beretta M.V., Schieppati M. (2007). Stance- and locomotion-dependent processing of vibration-induced proprioceptive inflow from multiple muscles in humans. J. Neurophysiol..

[46] Lee B.C., Martin B.J., Sienko K.H. (2012). Directional postural responses induced by vibrotactile stimulations applied to the torso. Exp. Brain Res..

[47] Sienko K.H., Balkwill M.D., Oddsson L.I.E., Wall C. (2013). The effect of vibrotactile feedback on postural sway during locomotor activities. J. Neuroeng. Rehabil..

[48] Curthoys I.S., Grant J.W., Pastras C.J., Brown D.J., Burgess A.M., Brichta A.M., Brichta A.M., Lim R. (2019). A review of mechanical and synaptic processes in otolith transduction of sound and vibration for clinical VEMP testing. J. Neurophysiol..

[49] Fabre C., Tan H., Dumas G., Giraud L., Perrin P., Schmerber S. (2021). Skull Vibration Induced Nystagmus Test: Correlations with Semicircular Canal and Otolith Asymmetries. Audiol. Res..

[50] Honegger F., van Spijker G.J., Allum J.H. (2012). Coordination of the head with respect to the trunk and pelvis in the roll and pitch planes during quiet stance. Neuroscience.

[51] Nanhoe-Mahabier W., Allum J.H., Pasman E.P., Overeem S., Bloem B.R. (2012). The effects of vibrotactile biofeedback training on trunk sway in Parkinson’s disease patients. Parkinsonism Relat. Disord..

[52] Bao T., Klatt B.N., Carender W.J., Kinnaird C., Alsubaie S., Whitney S.L., Sienko K.H. (2019). Effects of long-term vestibular rehabilitation therapy with vibrotactile sensory augmentation for people with unilateral vestibular disorders—A randomized preliminary study. J. Vestib. Res..

[53] Goebel J.A., Sinks B.C., Parker B.E.J., Richardson N.T., Olowin A.B., Cholewiak R.W. (2009). Effectiveness of head-mounted vibrotactile stimulation in subjects with bilateral vestibular loss: A phase 1 clinical trial. Otol. Neurotol..

